# Non-pharmaceutical primary care interventions to improve mental health in deprived populations: a systematic review

**DOI:** 10.3399/BJGP.2022.0343

**Published:** 2023-02-07

**Authors:** Louise M Tanner, Josephine M Wildman, Akvile Stoniute, Madeleine Still, Kate Bernard, Rhiannon Green, Claire H Eastaugh, Katie H Thomson, Sarah Sowden

**Affiliations:** Population Health Sciences Institute, Newcastle University; National Institute for Health and Care Research (NIHR) Innovation Observatory, Newcastle University, Newcastle-upon-Tyne.; NIHR/Health Education England Integrated Clinical Academic clinical lecturer and honorary consultant in public health, Population Health Sciences Institute, Newcastle University; NIHR Applied Research Collaboration North East and North Cumbria, Newcastle-upon-Tyne.; Population Health Sciences Institute, Newcastle University; National Institute for Health and Care Research (NIHR) Innovation Observatory, Newcastle University, Newcastle-upon-Tyne.; Population Health Sciences Institute, Newcastle University; National Institute for Health and Care Research (NIHR) Innovation Observatory, Newcastle University, Newcastle-upon-Tyne.; Population Health Sciences Institute, Newcastle University, Newcastle-upon-Tyne.; NIHR Innovation Observatory, Newcastle University, Newcastle-upon-Tyne.; Population Health Sciences Institute, Newcastle University; National Institute for Health and Care Research (NIHR) Innovation Observatory, Newcastle University, Newcastle-upon-Tyne.; NIHR/Health Education England Integrated Clinical Academic clinical lecturer and honorary consultant in public health, Population Health Sciences Institute, Newcastle University; NIHR Applied Research Collaboration North East and North Cumbria, Newcastle-upon-Tyne.; NIHR/Health Education England Integrated Clinical Academic clinical lecturer and honorary consultant in public health, Population Health Sciences Institute, Newcastle University; NIHR Applied Research Collaboration North East and North Cumbria, Newcastle-upon-Tyne.

**Keywords:** healthcare disparities, health inequalities, systematic review, mental disorders, primary health care, socioeconomic factors

## Abstract

**Background:**

Common mental health disorders are especially prevalent among people from socioeconomically disadvantaged backgrounds. Non-pharmaceutical primary care interventions, such as social prescribing and collaborative care, provide alternatives to pharmaceutical treatments for common mental health disorders, but little is known about the impact of these interventions for patients who are socioeconomically disadvantaged.

**Aim:**

To synthesise evidence for the effects of non-pharmaceutical primary care interventions on common mental health disorders and associated socioeconomic inequalities.

**Design and setting:**

Systematic review of quantitative primary studies published in English and undertaken in high-income countries.

**Method:**

Six bibliographic databases were searched and additional grey literature sources screened. Data were extracted onto a standardised proforma and quality assessed using the Effective Public Health Practice Project tool. Data were synthesised narratively and effect direction plots were produced for each outcome.

**Results:**

Thirteen studies were included. Social-prescribing interventions were evaluated in 10 studies, collaborative care in two studies, and a new model of care in one study. Positive results (based on effect direction) were reported for the impact of the interventions on wellbeing in groups that were socioeconomically deprived. Inconsistent (mainly positive) results were reported for anxiety and depression. One study reported that people from the group with least deprivation, compared with the group with greatest deprivation, benefitted most from these interventions. Overall, study quality was weak.

**Conclusion:**

Targeting non-pharmaceutical primary care interventions at areas of socioeconomic deprivation may help to reduce inequalities in mental health outcomes. However, only tentative conclusions can be drawn from the evidence in this review and more-robust research is required.

## INTRODUCTION

Globally, most patients with a common mental disorder, such as depressive and anxiety disorders, are seen only in primary care.^1^ In England in 2008, the Improving Access to Psychological Therapies (IAPT) service, which is part of the NHS, embedded psychological ‘talking’ therapies into primary care and, since then, an expanding range of non-pharmaceutical interventions are increasingly being offered as alternative forms of care for patients with common mental health disorders who present to primary care. As an example, healthcare systems as diverse as those of the UK and the US are introducing new models of personalised and collaborative care, such as social-prescribing interventions that link patients with sources of community support and clinical psychologists who are embedded into primary care.^2^^,^^3^

Common mental health disorders are most prevalent in people experiencing socioeconomic disadvantage and there is concern among primary care practitioners that an overreliance on psychotropic medications risks medicalising everyday stresses and the distress caused by poverty.^4^ In the UK and other high-income countries, addressing both mental ill health and health inequalities are pressing policy goals.^5^ There is a need for evidence about which non-pharmaceutical primary care interventions — such as social prescribing and new models of care — are effective at improving common mental health disorders in patients living in socioeconomic deprivation, and whether these interventions reduce, or potentially increase, health inequalities. A recent systematic review and meta-analysis of the IAPT service found improvements in depression and anxiety;^6^ however, there is evidence that patients who are socioeconomically disadvantaged struggle to access IAPT services.^7^^–^^9^ Focusing on patient groups that are socioeconomically disadvantaged, this systematic review is, to the authors’ knowledge, the first to review the impact of a range of alternative non-pharmaceutical interventions delivered in primary care on common mental health disorder-related outcomes.

## METHOD

The methods are described in detail in the published protocol^10^ and summarised here. The protocol was also registered in PROSPERO (registration number: CRD42021281166). This review was undertaken following the Preferred Reporting Items for Systematic Reviews and Meta-Analyses Equity (PRISMA-E) guidelines;^11^ in line with those PRISMA-E guidelines, a framework was developed in the protocol,^10^ outlining the pathway between the intervention and mental health outcomes in relation to social inequalities.

**Table 1:** How this fits in

New models of health care and clinical practice, such as social prescribing and collaborative care, are increasingly used as non-pharmaceutical alternatives for treating common mental disorders in primary care. However, there is a lack of evidence available to GPs about the effectiveness of these types of interventions for patients who are socioeconomically disadvantaged, among whom common mental health disorders are most prevalent. This systematic review synthesised the international evidence, exploring the impact on common mental health disorder outcomes for patients who are socioeconomically disadvantaged. There was evidence for an overall positive effect on anxiety, depression, self-reported mental health, and wellbeing; however, the evidence base was weak.

### Research questions

The research questions for this review of quantitative evidence, incorporating the population, intervention, comparators, and outcomes (PICO), were:

- which non-pharmaceutical primary care interventions improve common mental health disorder-related outcomes among people from socioeconomically disadvantaged backgrounds compared with no, or an alternative, intervention? And
- which non-pharmaceutical primary care interventions reduce inequalities in common mental health disorder-related adverse health outcomes between the least and most socioeconomically disadvantaged backgrounds?

### Literature search

The following bibliographic databases were searched from inception until 1 June 2021: MEDLINE, Applied Social Sciences Index and Abstracts, CINAHL, Embase, PsycINFO, and Scopus. Grey literature was identified from the Social Prescribing Network and the Social Interventions Research and Evaluation Network. Citation chaining of relevant systematic reviews and from the reference lists of included studies was additionally undertaken. The search strategy used in MEDLINE is available in Supplementary Appendix S1.

### Study selection

Titles and abstracts were screened in Rayyan^12^ to identify relevant studies; full texts of potentially relevant studies were sourced and assessed for eligibility, using the criteria summarised in Supplementary Box S1. Socioeconomic disadvantage was defined based on aggregate area-level indicators (for example, the Index of Multiple Deprivation [IMD]) or individual-level characteristics (for example, unemployment rate within the sample). One reviewer screened each record, and a second reviewer checked a random 10% sample at both stages of the screening process. Considerable time was spent, as a group, discussing the eligibility criteria to ensure these were applied; any articles for which there was no clear-cut screening decision were flagged to the team for consensus. Screening conflicts were resolved via discussion among the research team.

### Data extraction

The following data were extracted: citation details, study characteristics, intervention/control group characteristics, intervention details, comparators, outcomes, analysis, and results. These data were placed into a spreadsheet using Microsoft Excel and 10% were checked by a second reviewer.

### Quality appraisal

The quality of each study was assessed using the Effective Public Health Practice Project Quality Assessment Tool for Quantitative Studies.^13^ This tool was chosen because it can be applied across a range of different quantitative study designs. Each study was assessed independently by a single reviewer, with 10% of quality appraisals checked by a second reviewer.

### Data synthesis

Meta-analysis was not feasible due to heterogeneity in study designs, population characteristics, and outcomes assessed. Narrative synthesis was used alongside effect direction plots.^14^ The results were synthesised according to outcome type (that is, anxiety and depression, distress, wellbeing, self-reported mental health, and healthcare utilisation for common mental health disorders). For continuous measures, pre- and post-intervention mean values (for example, mean anxiety) for within-group studies were compared; post-intervention scores (adjusted for baseline values) between the intervention and control groups were compared for between-group studies. For binary outcomes, outcome rates at baseline versus post-intervention were compared for within-group studies, and post-intervention outcome rates (adjusted for baseline values) between the intervention and control groups were compared for between-group studies, where the data were available.

## RESULTS

### Study identification

**Figure 1.**
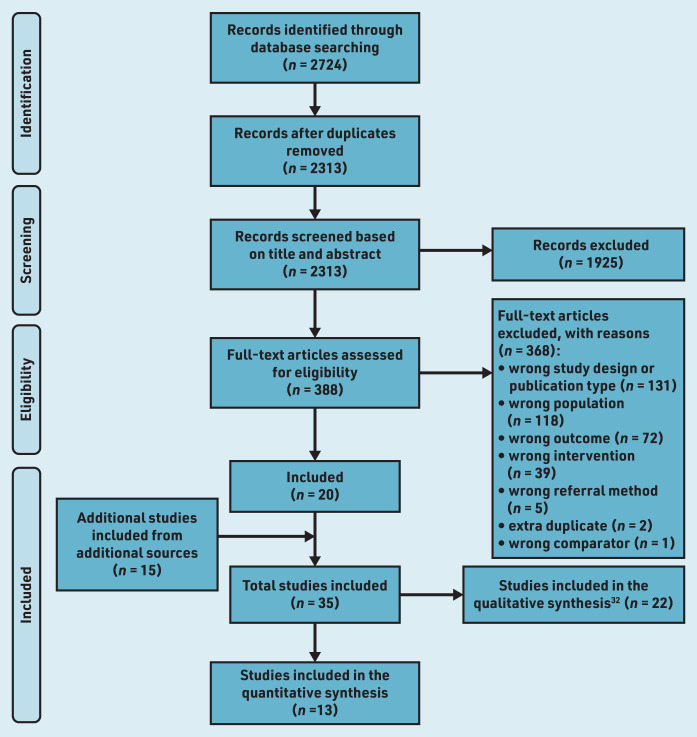
*PRISMA flowchart of study selection process.*

Thirteen studies^15^^–^^27^ were included in the narrative synthesis and effect direction plots (see Supplementary Table S1). Figure 1 illustrates the flow of included and excluded studies.

### Study characteristics

Included study designs comprised three randomised controlled trials (RCTs),^16^^,^^22^^,^^24^ one non-randomised controlled trial,^21^ one cohort study with a between-groups design,^25^ and eight cohort studies with a before–after, within-groups design.^15^^,^^17^^–^^20^^,^^23^^,^^26^^,^^27^ Nine studies^16^^–^^20^^,^^23^^–^^26^ were from England, two^21^^,^^22^ from Scotland, one^27^ from Canada, and one^15^ from Australia. Ten studies^15^^,^^17^^–^^23^^,^^25^^,^^27^ reported outcomes from a range of social-prescribing interventions. Two^16^^,^^24^ reported on collaborative care interventions, using non-medical care managers working with a patient’s clinician to support condition management and improve outcomes. A further study^26^ reported on a new model of care that combined aspects of social prescribing, social action, and community linkage.

Most of the interventions were targeted at people from socioeconomically deprived backgrounds, defined using area-level^16^^–^^18^^,^^20^^,^^22^^,^^23^^,^^25^^,^^26^ or individual-level^15^^,^^19^^,^^21^^,^^24^ measures (summarised in Supplementary Table S1). A further study^27^ indicated that participants were from low-income backgrounds, but it was not clear if this was based on an area-level or individual-level measure of deprivation. Only one study^25^ delivered a universal intervention to participants from a range of socioeconomic backgrounds and compared the effects of the intervention between IMD quintiles.

### Quality assessment

Quality assessment scores are summarised in Supplementary Box S2. One RCT^16^ received a global ‘strong’ quality assessment rating and one study^24^ received a global ‘moderate’ rating; the remainder received a global ‘weak’ overall rating.

### Effectiveness of the intervention

The characteristics, main results, and direction of effect from the 13 included studies are presented in Supplementary Table S1 and the following narrative synthesis.

#### Anxiety and depression

Four studies — comprising three cluster RCTs^16^^,^^22^^,^^24^ and one single-arm, before-and-after cohort study^20^ — reported effects of non-pharmaceutical primary care interventions on anxiety. The interventions included collaborative care^16^^,^^24^ and social prescribing.^20^^,^^22^ Three of the studies^16^^,^^20^^,^^24^ reported positive effects of the interventions on reducing anxiety (based on direction of effect). The fourth study^22^ reported that people who were referred to a community link practitioner experienced reduced anxiety (adjusted for baseline levels) when compared with a control group that received usual care. However, the benefit to the intervention group was dose dependent: results showed a reduction in anxiety relative to the usual-care group for one and ≥3 meetings with the community link practitioner, but an increase in anxiety for two meetings.

Five studies^16^^,^^20^^,^^22^^,^^24^^,^^26^ reported effects of non-pharmaceutical primary care interventions on depression; these comprised three cluster RCTs^16^^,^^22^^,^^24^ and two single-arm, before-and-after cohort studies.^20^^,^^26^ The interventions involved social prescribing,^20^^,^^22^ collaborative care,^16^^,^^24^ and a new model of care.^26^ Four studies^16^^,^^20^^,^^24^^,^^26^ reported a reduction in depression associated with the intervention; the fifth study^22^ reported mixed or conflicting findings, including a positive effect (reduction in depression compared with the control group) after meeting the community link practitioner once or ≥3 times, but a negative effect (increase in depression compared with the control arm) after being referred to, and seeing, a community link practitioner twice.

The authors of a cohort study^18^ (in which one group was assessed pre- and post-intervention) reported that users of a social-prescribing service in an area of socioeconomic deprivation showed reductions in anxiety/depression according to the EuroQol-5D anxiety and depression subscale. For the purposes of the review reported here, this was classed as an inconclusive result as the study only reported the proportions of participants who had a reduction in anxiety/depression and did not report the proportions of those participants whose anxiety/depression was unchanged or worsened following the intervention.

#### Measures of distress

One single-arm, before-and-after cohort study,^15^ comprising a sample of participants who were mostly unemployed, reported a reduction (positive outcome) in mean distress among recipients of a social-prescribing intervention.

#### Wellbeing

Five cohort studies^17^^,^^18^^,^^23^^,^^25^^,^^26^ quantified the effects of non-pharmaceutical primary care interventions on wellbeing, assessed using either the Warwick–Edinburgh Mental Wellbeing Scale or the short-form version of the tool. Four of these^17^^,^^18^^,^^23^^,^^26^ were single-arm, before-and-after studies, and one^25^ was a between-group study. Four of the studies^17^^,^^18^^,^^23^^,^^25^ evaluated social-prescribing interventions and one study^26^ included a multicomponent new model of care intervention. Four of the studies^17^^,^^18^^,^^23^^,^^26^ reported improvements in wellbeing based on direction of effect; the fifth study^25^ reported a lower rate of improved wellbeing among individuals in the IMD quintile of greatest deprivation compared with those in the IMD quintile of least deprivation.

#### Self-reported mental health

One single-arm, before-and-after cohort study^27^ assessed the effects of a social-prescribing intervention on self-reported mental health in participants, nearly half of whom were from low-income backgrounds. An improvement in self-reported mental health was reported following the intervention.

#### Healthcare utilisation for common mental health disorders

Two studies^19^^,^^21^ reported results regarding the effects of social prescribing on healthcare utilisation for common mental health disorders; both reported inconsistent (positive and negative) results.

## DISCUSSION

### Summary

This systematic review summarises the available quantitative evidence for the effects of social prescribing, collaborative care, and new models of care interventions on outcomes for patients with common mental health disorders who experience socioeconomic deprivation. A total of 13 studies were identified that reported data addressing the impacts on common mental health disorders among patients who were socially disadvantaged.

Results were positive overall, but outcomes were not consistent where multiple studies contributed data. For anxiety and depression, all but one study reported positive results — the one that did not report a dose-dependent effect. Inconclusive results were also reported in relation to anxiety and depression combined. The studies reporting effects on distress and self-reported mental health both indicated a positive effect. For wellbeing, four studies reported a positive outcome. The results were inconsistent for healthcare utilisation, with both studies that looked at this reporting mixed results.

Only one study addressed the impacts of interventions on socioeconomic inequalities and common mental health disorders; it suggested that participants from areas of greatest deprivation were less likely to respond to the intervention compared with those from areas of least deprivation.

### Strengths and limitations

To the authors’ knowledge, this is the first review to synthesise evidence on the impact of interventions such as social prescribing, collaborative care, and new models of care on common mental health disorder-related outcomes for patients who are socioeconomically disadvantaged. The searches for this review included bibliographic databases, grey literature sources, and citation chaining. Bibliographic searches were undertaken on 1 June 2021 and it is possible that new studies meeting the inclusion criteria have been published since the searches were undertaken; however, due to the low-quality evidence included in this review, the authors feel it is unlikely that new evidence would drastically change the findings.

Effect direction plots were used, but these are not able to produce precise effect estimates and do not consider statistical significance. In addition, the authors intended to synthesise evidence on different types of inequality in relation to the PROGRESS-Plus dimensions as described in the protocol,^28^ but only had the resources to focus on one dimension of disadvantage (socioeconomic status).

The authors’ ability to address the first research question (which non-pharmaceutical primary care interventions improve common mental health disorder-related outcomes in socially disadvantaged communities?) was limited because the positive effects of the interventions were likely to have been inflated due to the low-quality evidence. Also, the heterogeneous nature of the evidence on the topic prevented statistical pooling of the data to derive an overall effect estimate. It was also not possible to properly address the second research question regarding the effects of non-pharmaceutical primary care interventions on common mental health disorder-related inequalities between people from the least and most socioeconomically disadvantaged backgrounds, due to limited primary evidence on this topic.

Most of the included studies used validated outcome assessment tools, but the main limitation was the inability to blind participants to their intervention status; coupled with the self-reported nature of the outcome assessments, this could introduce bias into the results. Many studies also either reported non-significant effects of the interventions or significance was not reported. In addition, for most studies, area-level measures were used to assess socioeconomic deprivation; this is potentially ecologically fallacious, as the samples were likely to include participants from non-socioeconomically deprived backgrounds. This potentially limits the generalisability of the findings, as does the fact that most of the studies included in the review were conducted in the UK.

Most interventions in this review were targeted at people living in areas of socioeconomic deprivation, an approach that enabled the authors to assess the effects of the interventions on those most at risk of experiencing a common mental health disorder. Only one study included participants from mixed socioeconomic backgrounds. Previous research has demonstrated that the delivery of universal interventions, proportionately applied to the most in need, are likely to reduce health inequalities across the whole population; targeted interventions that are delivered to the most disadvantaged groups, however, may raise the health of those targeted, but do not improve the health of those in the middle of the health inequalities spectrum.^29^^,^^30^

### Comparison with existing literature

Although non-pharmaceutical mental health interventions are increasingly being offered to primary care patients in communities that are socioeconomically disadvantaged, there is little robust published evidence regarding their effectiveness within this population. A recent review of IAPT services found that, although effective for some patients, the services often failed to reach patients with complex presentations, including socioeconomic disadvantage.^6^ A recent review of social-prescribing interventions found evidence of effectiveness in improving outcomes such as anxiety and depression; however, there was no evaluation of differential impacts on patients who were socioeconomically disadvantaged.^31^

With any intervention, there is a risk that patients with a low socioeconomic status will struggle with access. One study included in this review indicated that people from the areas of least deprivation benefited most from an intervention, which may result in increased health inequalities. A qualitative review conducted alongside this quantitative review also found that socioeconomic factors (for example, low income) were key barriers to accessing and engaging with interventions.^32^

### Implications for practice and research

Much of the evidence was weak and further investigation involving more-robust study designs (that is, between-group studies with larger sample sizes, more-objective outcome measures, and statistical-significance testing) is required. Given the intersectionality of disadvantage, further research is also needed to extend the focus of this review to other PROGRESS-Plus dimensions, and to explore the implications of multiple and overlapping layers of disadvantage and inequality.

The increasing popularity of social prescribing, collaborative care, and new models of care interventions to address common mental health disorders and the focus on addressing health inequalities creates a pressing need for practitioners to have access to evidence regarding what works to improve outcomes for patients experiencing socioeconomic disadvantage. Overall, the results from this review indicated a positive effect of a range of non-pharmaceutical primary care interventions on common mental health disorders and related symptoms. However, as most of the included studies were rated as being of low overall quality, it is possible that the effectiveness of the interventions may have been over-or underestimated.

There is a need for higher-quality research that examines the differential effects of interventions on patients with greater and lesser socioeconomic advantage, and explores the other PROGRESS-Plus criteria and the interrelationships therein. By targeting interventions specifically at socioeconomically disadvantaged areas and individuals from socioeconomically disadvantaged areas and backgrounds, inequalities in common mental health disorder-related health outcomes could be reduced. It would also be informative to identify which components of the interventions (for example, speaking to a link worker, undertaking an activity, or socialising with others) are associated with a positive effect.
